# Label-Free Quantitative Proteomic Analysis Reveals the Effects of Biogenic Silver Nanoparticles on *Fusarium keratoplasticum* and Their Therapeutic Potential in *Galleria mellonella* Larvae

**DOI:** 10.1021/acsomega.5c03275

**Published:** 2025-08-14

**Authors:** Glaucia Rigotto-Caruso, Aaron Curtis, Kevin Kavanagh, Marcia Regina von Zeska Kress

**Affiliations:** † Department of Clinical Analyses, Toxicology, and Food Science, School of Pharmaceutical Sciences of Ribeirão Preto, 28133 University of São Paulo (USP), Avenida do Café, s/n, CEP, 14040-903 Ribeirão Preto, São Paulo, Brazil; ‡ Department of Biology, 8798 Maynooth University, National University of Ireland, Maynooth, Co. Kildare W23 F2H6, Ireland

## Abstract

Antifungal drug resistance is a growing concern, necessitating new therapeutic alternatives. This study evaluated the antifungal activity and molecular effects of biogenic silver nanoparticles (AgNPs) synthesized using the culture filtrate of *Epicoccum nigrum* against *Fusarium keratoplasticum*, a highly resistant fungal species. AgNPs exhibited strong antifungal activity, with a MIC_50_ of 1.79 μg/mL and 92.85% growth inhibition at 5.92 μg/mL. Label-free quantitative proteomic analysis (LFQ-MS) revealed 52 proteins with significantly altered abundance after AgNP treatment, affecting the oxidative stress response, mitochondrial function, and riboflavin biosynthesis. Decreased levels of proteins involved in riboflavin biosynthesis and electron transport suggest metabolic and energy disruption, while increased levels of oxidative stress response and heat shock proteins indicate fungal stress. To assess toxicity and antifungal efficacy in vivo, *Galleria mellonella* larvae were exposed to AgNPs at 2.58 mg/kg, showing a 90% survival rate after 7 days. Hemocyte density increased temporarily with no long-term immune disruption. Proteomic analysis of hemolymph revealed minor protein abundance changes, mostly related to the immune response and metabolism. In fungal infection assays, larvae infected with *F. keratoplasticum* (10^5^ conidia/mL) had a 90% mortality rate, but AgNP treatment increased survival 5-fold (50% by day seven). These findings confirm that biogenic AgNPs act through the induction of oxidative stress, metabolic disruption, and mitochondrial damage in *F. keratoplasticum*. The combination of proteomic and in vivo data supports their efficacy and safety. Further studies should explore long-term toxicity and potential applications in medicine and agriculture to combat antifungal resistance.

## Graphical abstract

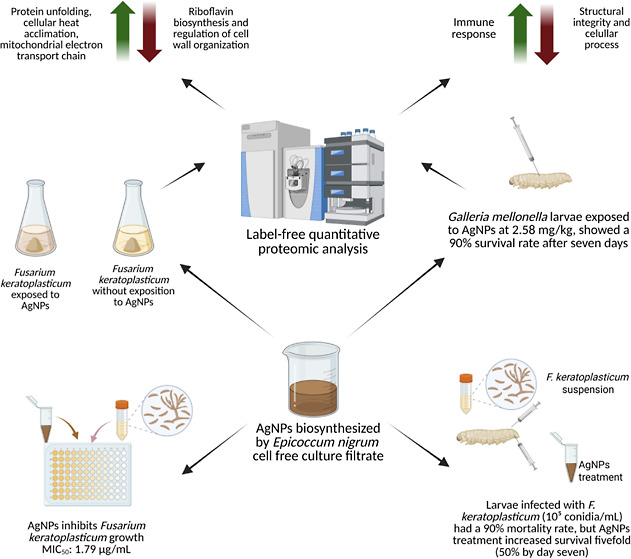

## Introduction

The global rise in antimicrobial resistance has emerged as one of the most pressing public health challenges, with fungal infections contributing significantly due to their high morbidity and mortality rates.^1,2^ Fungal pathogens are responsible for numerous infections, particularly among immunosuppressed patients, due to underlying diseases, medication regimens, or coinfections with bacteria or viruses. Even naturally infected immunocompetent individuals can be affected. The COVID-19 pandemic has further highlighted this problem, with a significant increase in invasive fungal infections among hospitalized patients.^3^ In response to the global threat posed by fungal pathogens, the World Health Organization published its first list of “priority fungal pathogens” in October 2022. The list includes 19 fungi that present a higher risk to public health, with the genus *Fusarium* classified as “high priority”.^4^

*Fusarium* spp. are widely known for causing significant damage and losses in agriculture, but human cases of fusariosis have also increased in recent years. At least 70 species can infect humans, mostly belonging to the *Fusarium solani* species complex (*Fusarium* solani species complex), which is responsible for up to 50% of severe infections.^5,6^ *Fusarium keratoplasticum* belongs to this complex and is often associated with superficial and systemic infections in humans. It has been identified in cases of keratitis, onychomycosis, and disseminated fusariosis, particularly among immunocompromised individuals, and exhibits high levels of intrinsic resistance to conventional antifungal therapies.^7,8^ Clinical isolates of *F. keratoplasticum* have also been recovered from hospital environments and shown to tolerate antifungal agents through both intrinsic and acquired mechanisms, including the overexpression of drug efflux transporters.^9^ Its ability to cause invasive disease and adapt to host-like conditions makes it a relevant target for therapeutic development.

This inherent resistance, coupled with the growing resistance to existing antifungals, demands further research and the development of novel molecules and drugs with antimicrobial action to create treatments that effectively control and eliminate fungal infections.^10,11^ In this context, nanotechnology offers a promising avenue for addressing this challenge. Silver nanoparticles (AgNPs) have gained significant attention in the biomedical field due to their broad-spectrum antimicrobial activity and potential to overcome microbial resistance.^12,13^ AgNPs exhibit distinct physical and chemical characteristics from their macro-scale counterparts, which are largely attributed to their reduced size and high surface-to-volume ratio.^14,15^ Among the various biological sources used for AgNPs biosynthesis, *Epicoccum nigrum* has been previously reported as effective in producing nanoparticles with antifungal activity against multiple pathogens, including *Candida*, *Aspergillus*, and *Fusarium* species.^16^ AgNPs may exhibit different mechanisms of action depending on their size, shape, and synthesis method. These unspecific mechanisms make AgNPs less susceptible to resistance development compared to traditional antifungal drugs that typically target a single cellular process.^17,18^ Despite these advantages, the precise mechanisms by which AgNPs promote antifungal effects on *Fusarium* species remain poorly understood, particularly at the molecular level. This study aims to investigate the antifungal activity of biogenic AgNPs, which are biosynthesized by an extremophilic fungus, *E. nigrum*, against *F. keratoplasticum*. It focuses on the efficacy of these nanoparticles in inhibiting fungal growth and their impact on the fungal proteome.

In addition to in vitro proteomic analysis, we employed the*Galleria mellonella* larval model to evaluate the therapeutic potential of AgNPs in vivo. *G. mellonella* larvae have been increasingly used as a model organism for studying microbial pathogenesis and host immune responses, as well as for testing the in vivo efficacy and toxicity of antimicrobial agents.^19^ This model has gained relevance due to its practical advantages (e.g., low cost, easy inoculation, rapid results) and the presence of an innate immune system that shares structural and functional similarities with that of mammals, including phagocytic hemocytes and the activation of Toll and Imd signaling pathways involved in host defense.^20^ By using this model, we aimed at evaluating both the in vivo antifungal efficacy of AgNPs and their potential cytotoxicity, thereby providing a more comprehensive understanding of their therapeutic potential.

## Results

### Assessment of the Antifungal Activity of AgNPs against *F. keratoplasticum*

The AgNPs employed in this study were biosynthesized by using the cell-free culture filtrate of *E. nigrum*. Their diameters ranged from 2.66 to 30.10 nm, with an average size of 13.17 nm. The size distribution of 75% of the particles was on average 16.67 nm, and the size distribution of 25% of the particles was on average 9.45 nm. The AgNPs were spherical, monodispersed, and exhibit a negative zeta potential of −23.5 mV, indicating high stability due to electrostatic repulsion, which prevents aggregation.

The antifungal efficacy of biosynthesized AgNPs against *F. keratoplasticum* was evaluated. An inhibition curve for the growth of *F. keratoplasticum* was generated using AgNPs concentrations ranging from 0.00 to 5.92 μg/mL. The study determined that the minimum inhibitory concentration required to inhibit 50% growth (MIC_50_) was 1.79 μg/mL, indicating significant antifungal activity at relatively low concentrations. Furthermore, at the maximum tested concentration of 5.92 μg/mL, a substantial inhibition of 92.85% was observed (Figure 1), demonstrating the potential of AgNPs as a strong antifungal agent against *F. keratoplasticum*.

**Figure 1:**
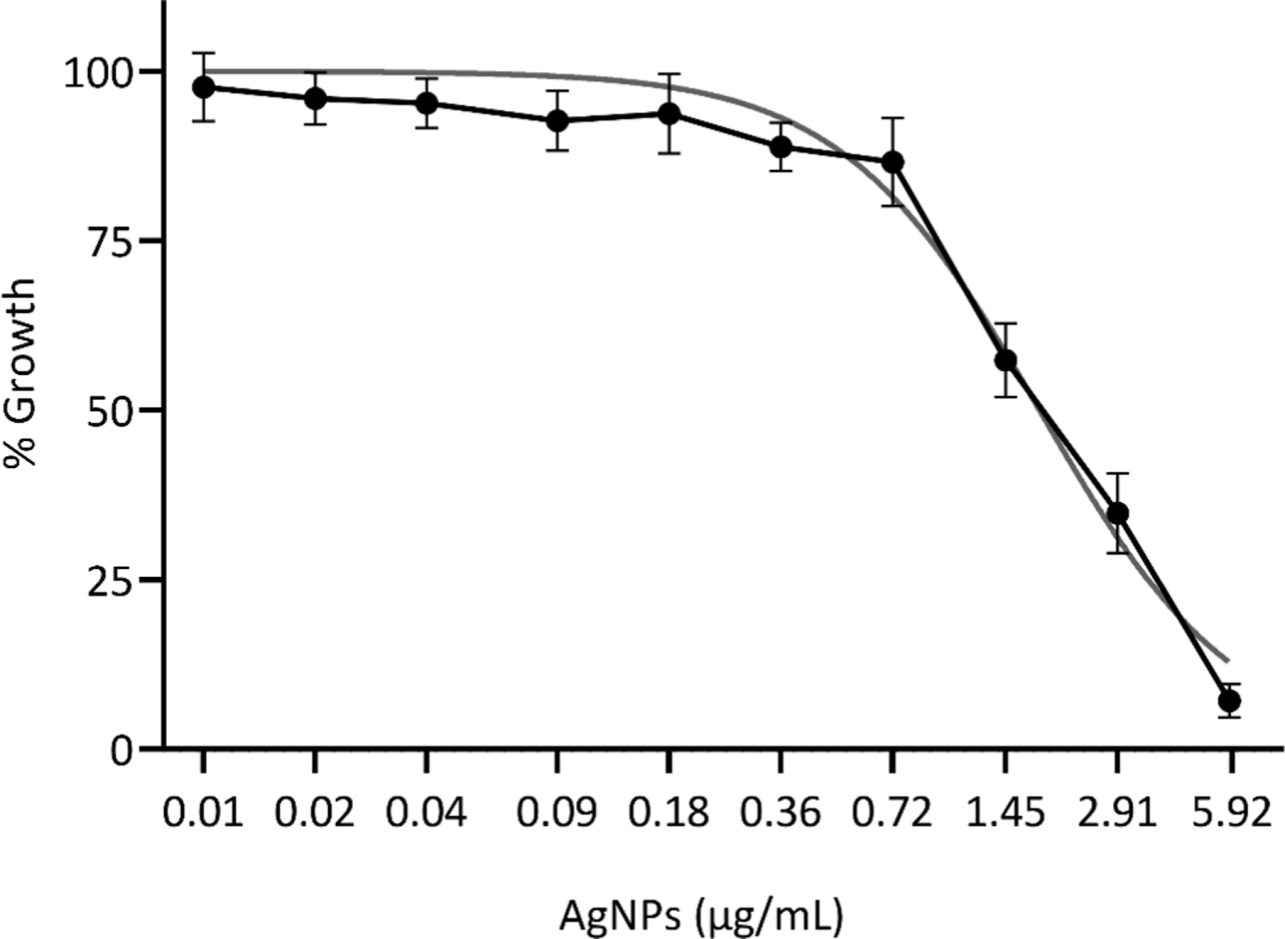
Dose-dependent inhibition of *F. keratoplasticum* growth by AgNPs. Data are from three independent experiments, with mean values and standard deviations shown for each data point. The experiments were conducted over three independent days, with a total of 11 replicates (*n* = 11).

### Effect of AgNPs on the Proteome of *F. keratoplasticum*

*F. keratoplasticum* was exposed to 1.79 μg/mL of AgNPs for 24 h, as determined from the inhibition growth curve. Proteins were extracted, purified, identified, and analyzed by using label-free quantitative mass spectrometry (LFQ-MS). Principal component analysis (PCA) of the proteomic data revealed a clear separation between the proteomes of control and AgNP-treated samples, indicating a distinct shift in the proteomic profile in response to AgNPs exposure (Figure 2A). A heat map was generated to illustrate these differences, demonstrating distinct abundance patterns across proteins (Figure 2B).

**Figure 2:**
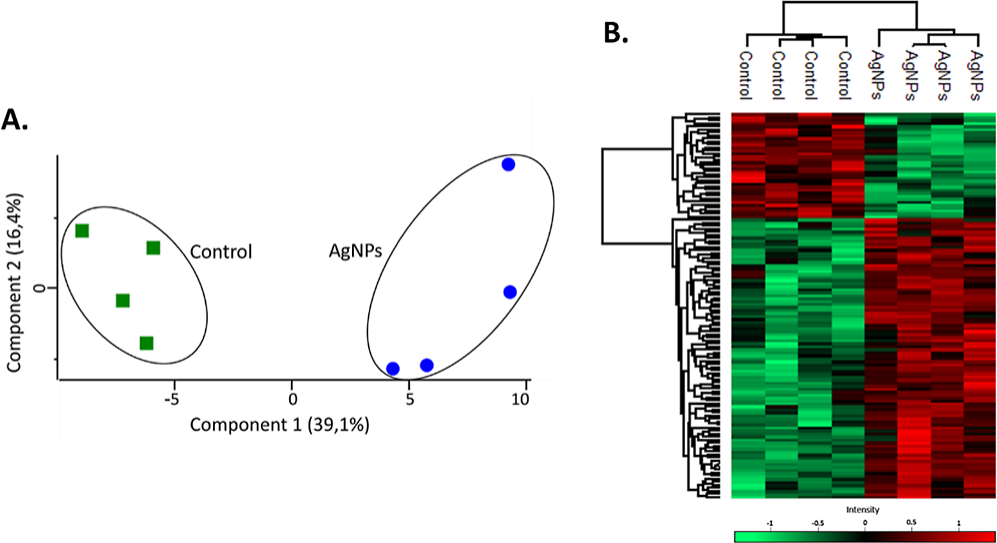
(A). PCA of the *F. keratoplasticum* proteome exposed to AgNPs (blue) and untreated control sample (green). Each point represents a replicate of the respective treatment. (B). Hierarchical clustering of the average abundance levels of proteins from the *F. keratoplasticum* proteome, untreated and treated with AgNPs. Columns represent each sample group, while rows correspond to proteins grouped into major clusters based on similar protein abundance patterns, with higher and lower abundance proteins indicated in red and green, respectively.

A total of 853 proteins were identified, of which 52 were considered statistically significant and differentially abundant (SSDA) between the untreated control and AgNPs-treated samples. Among the 52 significantly affected proteins, 30 exhibited increased abundance, while 26 showed a decrease in abundance compared with the untreated control. This visualization highlights the marked divergence in proteomic profiles between the untreated control and the AgNPs-treated groups.

A volcano plot was generated to visualize the distribution of all 853 identified proteins based on fold changes and statistical significance (Figure 3A). This plot highlights the proteins with altered abundance, demonstrating the impact of AgNPs exposure on the fungal proteome. Gene ontology (GO) analysis performed using STRING revealed that proteins with decreased abundance (Figure 3B) were associated with the riboflavin biosynthetic process (GO:0009231), regulation of cell wall organization or biogenesis (GO:1903338), and the ribonucleotide biosynthetic process (GO:0009260). These findings indicate that AgNPs may lead to dysregulation in cell wall organization and disrupt riboflavin biosynthesis, a precursor of flavin adenine dinucleotide (FAD) and flavin mononucleotide (FMN), which are critical for redox reactions as electron carriers. Conversely, proteins with increased abundance (Figure 3C) are associated with protein unfolding (GO:0043335), cellular heat acclimation (GO:0070370), and mitochondrial electron transport from cytochrome c to oxygen (GO:0006123), suggesting that AgNPs promote cellular stress and affect respiratory pathways.

**Figure 3:**
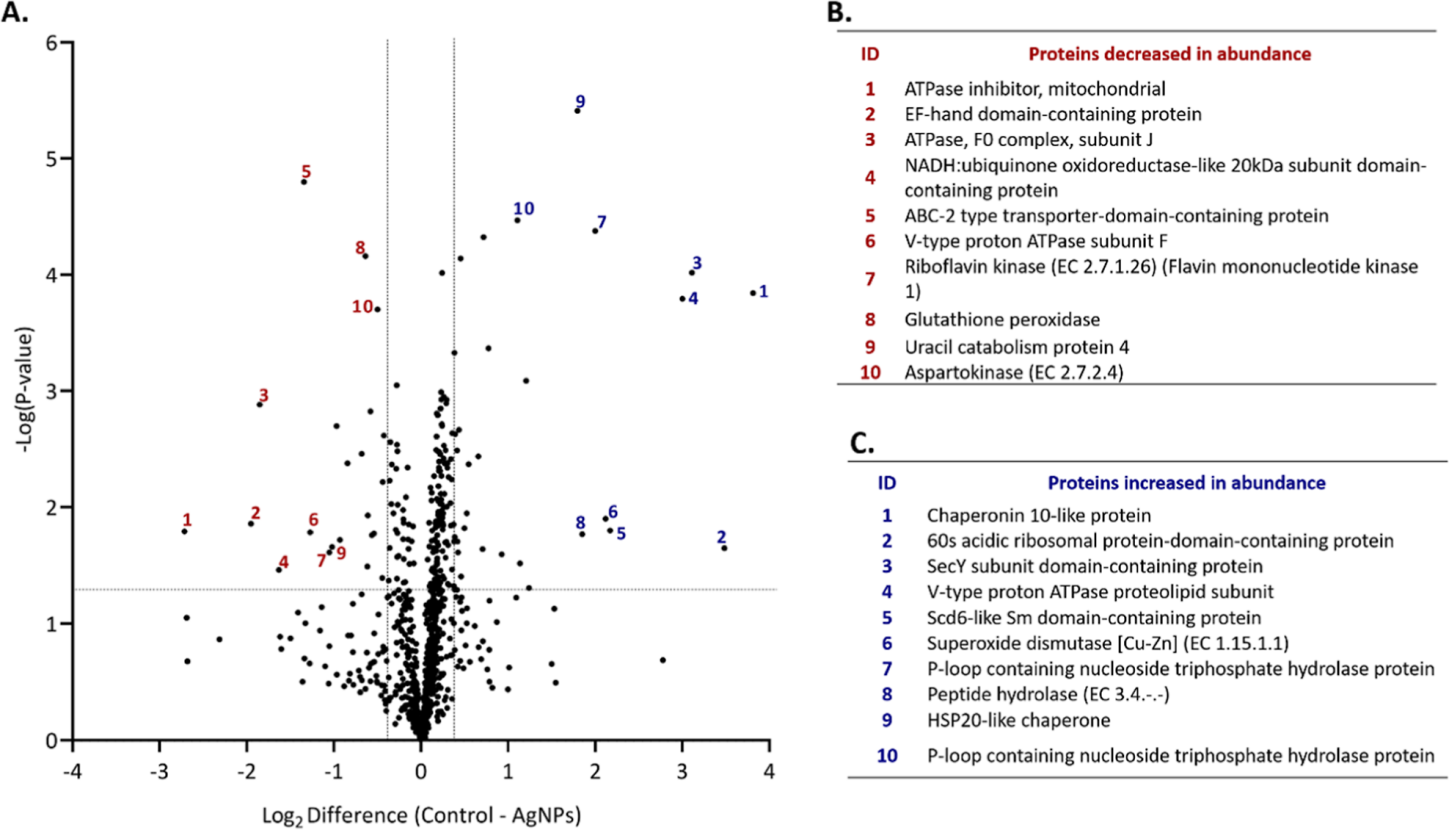
(A). Volcano plot of SSDA proteins in *F. keratoplasticum* treated with AgNPs (right side) and untreated control (left side). Pair-wise Student’s *t* tests (*P*-value < 0.05) were used to generate a volcano plot, showing how the quantified proteins are distributed based on their statistical significance (−log_10_ *P*-value) and the log_2_ difference in average LFQ intensity. Proteins considered statistically significant (*P*-value < 0.05) appear above the horizontal line, while those with notable changes in abundance are placed on either side of the vertical lines. (B). Names of 10 proteins decreased in abundance (red ID). (C). Names of 10 proteins increased in abundance (blue ID).

### AgNP Toxicity in *G. mellonella*

The toxicity of AgNPs was evaluated by using *G. mellonella* larvae. Two different doses of AgNPs were administered: 2.58 mg/kg and 1.29 mg/kg. The higher dose (2.58 mg/kg) resulted in a survival rate of 90%, while the lower dose (1.29 mg/kg) gave a 100% survival rate. The control groups, naïve and treated with phosphate-buffered saline (PBS) 1×, showed a 100% survival rate. These results suggest that neither concentration of AgNPs induced significant toxicity in *G. mellonella*, as reflected by the high survival rates observed in both treatment groups (Figure 4).

**Figure 4:**
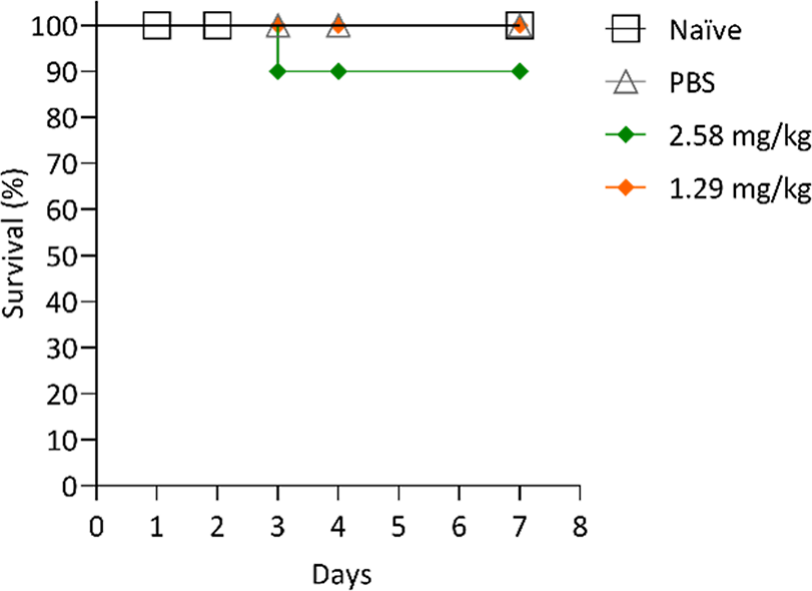
Survival curve of *G. mellonella* treated with AgNPs. The graph represents the survival rates of *G. mellonella* larvae over a period of 7 days following treatment with AgNPs. Larvae were injected with two doses: 2.58 mg/kg (green line) and 1.29 mg/kg (orange line). PBS 1× was used as a negative control (gray line).

To evaluate the impact of AgNPs on the cellular immune response, the hemocyte densities in the *G. mellonella* larvae were determined at various time points following exposure to 2.58 mg/kg of AgNPs. Hemocyte density increased from 2.37 × 10^6^ to 1.2 × 10^7^ hemocytes/mL within 1 h, indicating a robust cellular response. However, by 16 h, hemocyte density began to decrease, reaching 4.43 × 10^6^ hemocytes/mL at 48 h. The control group treated with PBS 1× maintained stable hemocyte densities throughout the 48 h period (Figure 5). In summary, exposure to AgNPs induced a transient increase in hemocyte density in *G. mellonella*, indicating an initial cellular response. However, hemocyte levels declined over time, suggesting that the larvae could modulate their immune response after AgNP exposure without prolonged disruption.

**Figure 5:**
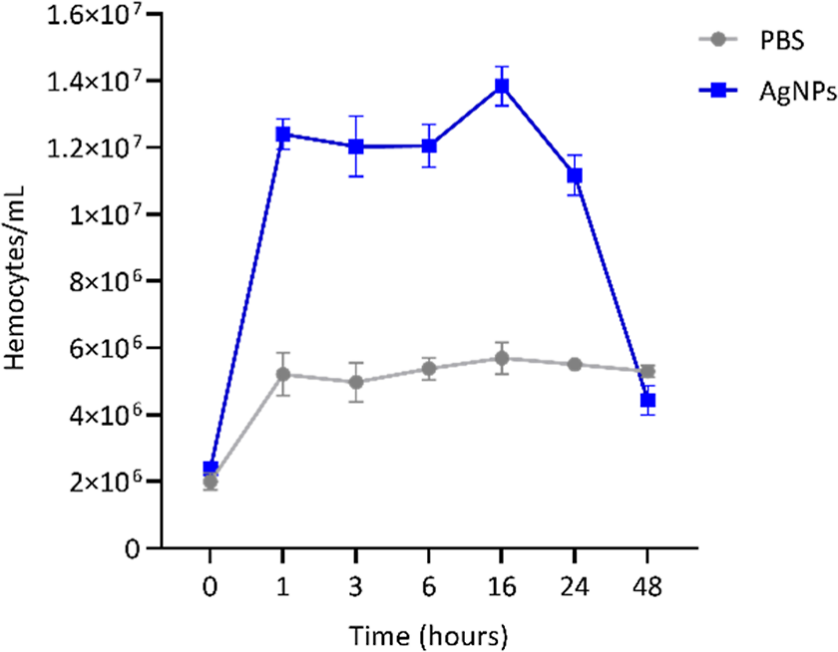
Hemocyte density in *G. mellonella* larvae. Groups of three larvae were injected with AgNPs (blue line) at a dose of 2.58 mg/kg, and hemocytes densities were ascertained at 1, 3, 6, 16, 24, and 48 h postinjection with AgNPs. As a control, hemocytes from larvae injected with only PBS 1× (gray line) were also counted over the same time points.

### Effect of AgNPs on *G. mellonella* Proteome

Label-free quantitative proteomic analysis of *G. mellonella* larvae treated with AgNPs revealed minimal changes in protein abundance compared with untreated control larvae. PCA (Figure 6A) showed clear separation between the treated and control groups, reflecting distinct differences in their proteomic profiles. Further analysis involved hierarchical clustering of Z-score normalized intensity values, revealing distinct protein clustering between the treated and control groups (Figure 6B).

**Figure 6:**
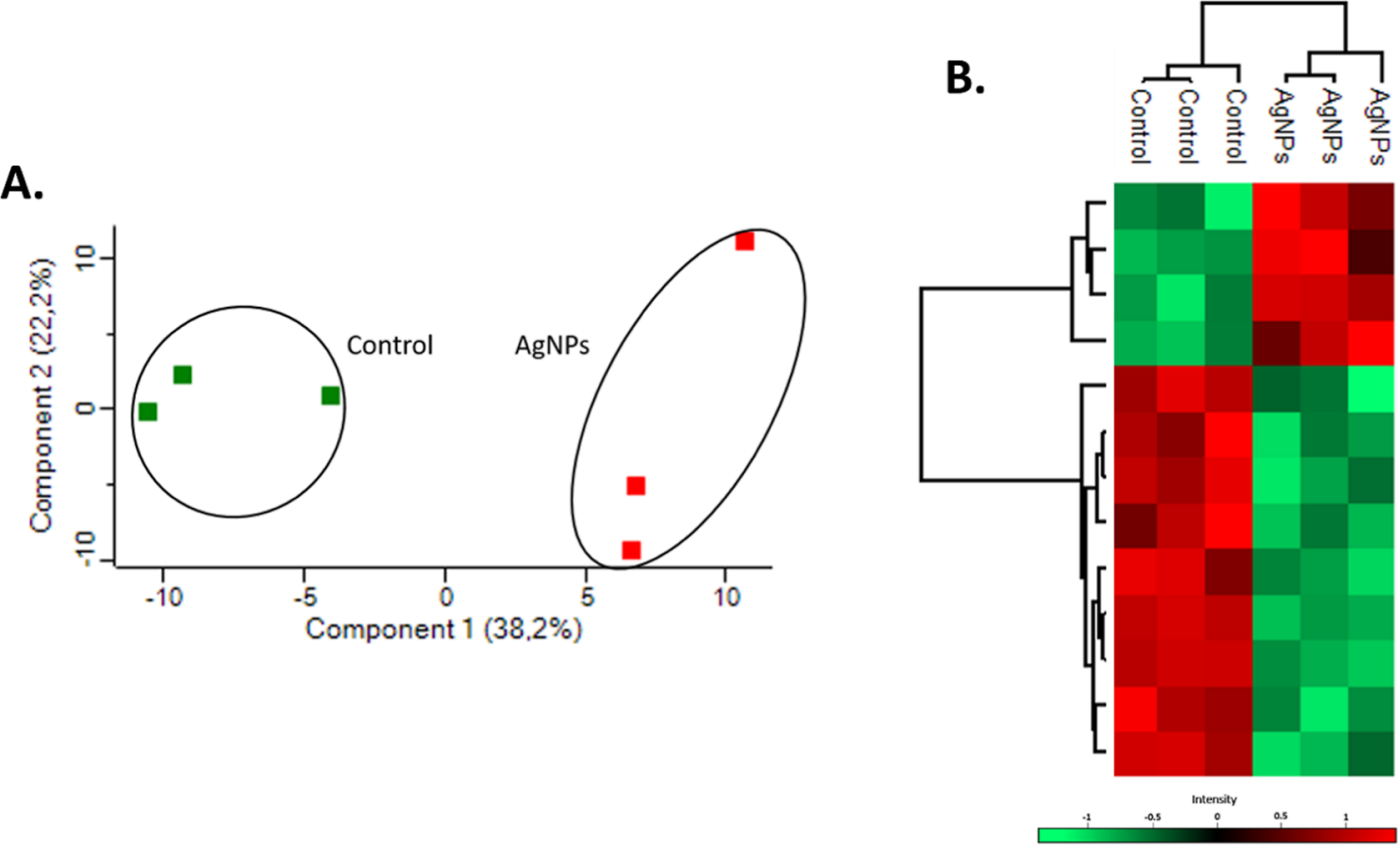
(A). PCA of the hemolymph proteome from *G. mellonella* larvae treated with AgNPs (red squares) and untreated control larvae (green squares). (B). Hierarchical clustering of the average abundance levels of proteins. Columns represent each sample group, while rows correspond to proteins grouped into major clusters based on similar protein abundance patterns. Higher and lower abundance proteins are indicated in red and green, respectively.

A total of 302 proteins were identified across the samples, of which 36 proteins showing statistically significant changes in abundance (ANOVA, *p* < 0.05). These proteins are visualized in volcano plot (Figure 7A), which highlights both the magnitude and significance of these differences. Proteins that exhibited reduced abundance following AgNPs exposure were multifunctional protein ADE2 (−4.59-fold), aminopeptidase W07G4.4 (−4.23-fold), arginine kinase (−4.15-fold), malate dehydrogenase (−4.04-fold), laminin subunit alpha-like (−3.54-fold), and nidogen (−3.07-fold). These proteins are linked to disruptions in metabolism, structural integrity, and cellular processes (Figure 7B). Conversely, several proteins exhibited increased abundance in response to AgNP exposure including glutamine synthetase (+9.13-fold), indicating heightened nitrogen metabolism; lipase 3-like (+3.36-fold), involved in lipid breakdown; cathepsin L (+1.78-fold) and peptidoglycan recognition protein (+1.41-fold) involved in immune response. Additionally, uncharacterized proteins corresponding to Uniprot IDs A0A6J1WTK1 (+5.19-fold), A0A6J3C9T0 (+1.89-fold), and A0A6J3CBP3 (+1.79-fold) were more abundant in the treated samples (Figure 7C).

**Figure 7:**
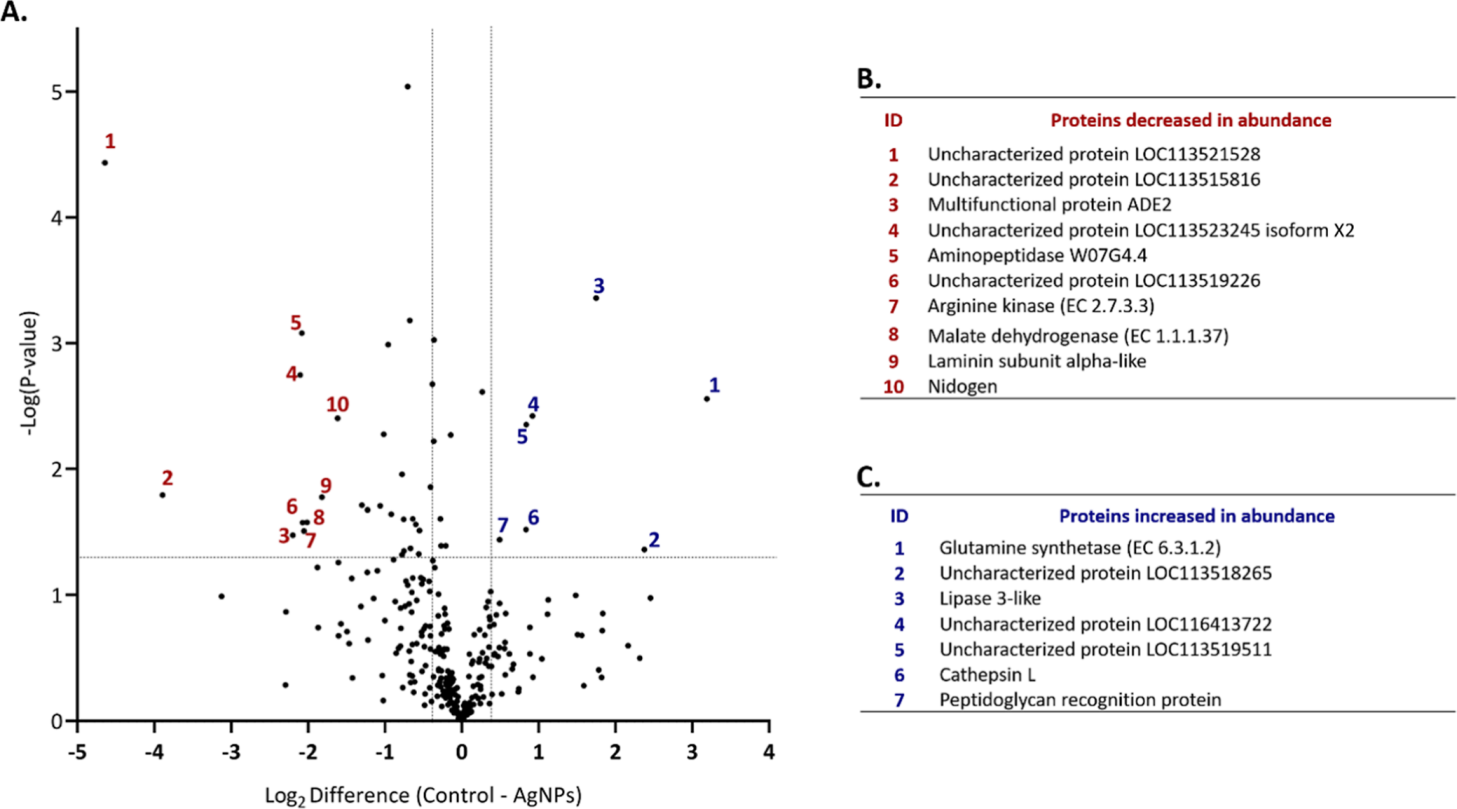
(A). Volcano plots of SSDA proteins in *G. mellonella* larvae treated with AgNPs at 2.58 mg/kg (right side) versus untreated control (left side). Pairwise Student’s *t* tests (*P*-value < 0.05) were applied to generate volcano plot, showing the distribution of quantified proteins based on *P*-value (−log_10_ *P*-value) and log_2_ mean LFQ intensity difference. Proteins above the horizontal line are statistically significant (*P*-value < 0.05), while proteins with significant changes in abundance are showed to the right/left of the vertical lines. (B). Names of proteins decreased in abundance (red ID). (C). Names of proteins increased in abundance (blue ID).

Further proteomic analysis revealed the presence of proteins associated with the innate immune response, suggesting that AgNPs may trigger immune-related signaling pathways in *G. mellonella*. STRING analysis identified 8 proteins linked to the KEGG pathway map04624, which corresponds to the Toll and Imd signaling pathways. Additionally, GO analysis revealed that 11 proteins are involved in the biological process “Response to external stimulus” (GO:0009605). These proteomic changes likely explain the heightened cellular response observed following AgNP exposure. Despite the presence of AgNP, no significant humoral response was detected, and the minimal disruption in protein abundance may account for the survival of *G. mellonella* under these conditions.

### Survival Analysis of *G. mellonella* Infected with *F. keratoplasticum*

To identify the effective infectious concentration for subsequent experiments, survival curve analysis was performed with *G. mellonella* larvae infected with different concentrations of *F. keratoplasticum* conidia (Figure 8). At a concentration of 10^4^ conidia/mL, 60% of the larvae survived over a 7 day period. In contrast, an inoculum of 10^5^ conidia/mL caused a sharp decline in survival, from 75% to 30% on day 4 to 0% by day 7. At a higher inoculum of 10^6^ conidia/mL, 100% mortality was observed by day 6, while the highest concentration of 10^7^ conidia/mL led to complete mortality by day 3. Based on these findings, the 10^5^ conidia/mL concentration was selected for subsequent infection of larvae. This concentration was used to evaluate whether AgNPs could effectively combat the infection.

**Figure 8:**
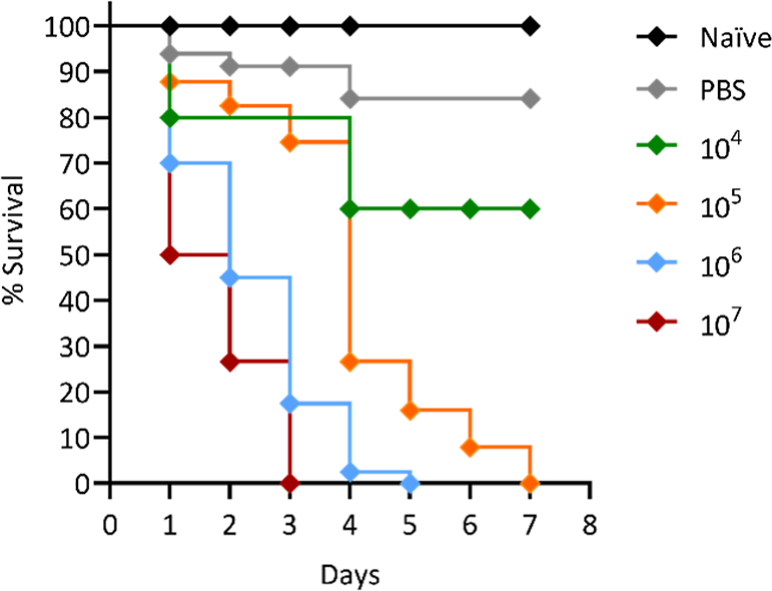
Survival curves of *G. mellonella* larvae infected with varying concentrations of *F. keratoplasticum*. The graph depicts the survival of *G. mellonella* larvae over 7 days postinfection with different inoculum concentrations: 10^4^ conidia/mL (green), 10^5^ conidia/mL (orange), 10^6^ conidia/mL (blue), and 10^7^ conidia/mL (red). Control groups include uninfected larvae (black) and larvae injected with PBS 1× (gray).

### Infection of *G. mellonella* with *F. keratoplasticum* and Treatment with AgNPs

The survival curves demonstrate that larvae infected with *F. keratoplasticum* at a concentration of 10^5^ conidia/mL experienced only a 10% survival rate by the seventh day (Figure 9). In contrast, larvae treated with AgNPs 1 h after inoculation showed a big improvement in survival, with 50% of the larvae surviving by day 7. Notably, treatment with AgNPs alone did not cause any mortality in the larvae. Furthermore, both PBS-treated larvae and untreated control groups showed no significant mortality, indicating that neither PBS nor the absence of treatment affected larval survival.

**Figure 9:**
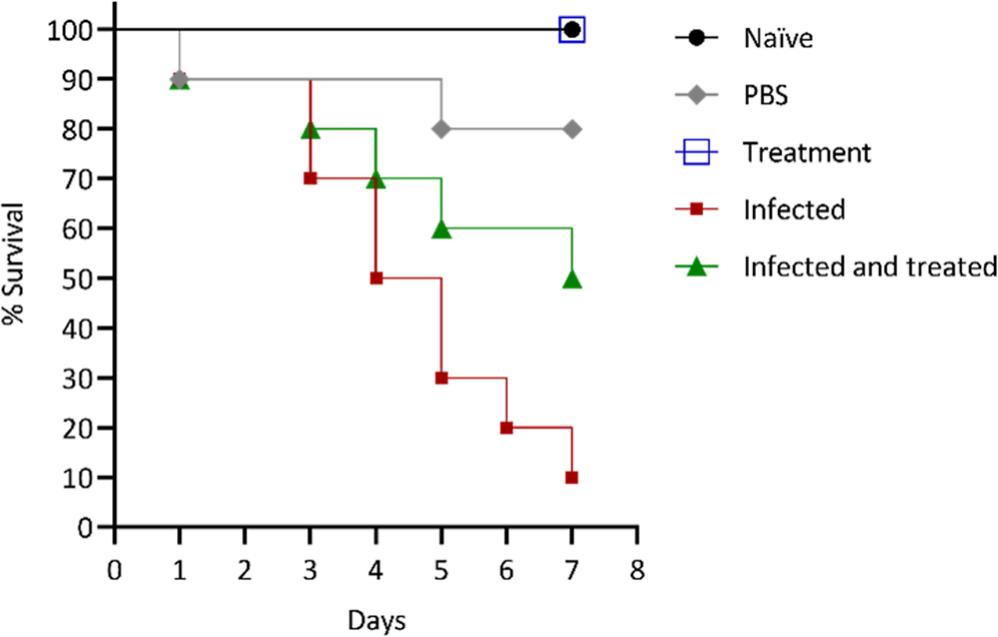
Effect of AgNPs on the survival of *G. mellonella* larvae infected with *F. keratoplasticum*. The graph shows the survival of *G. mellonella* larvae over 7 days postinfection with 10^5^ conidia/mL of *F. keratoplasticum* (red), AgNP treatment alone (blue), and a combination of 10^5^ conidia/mL of *F. keratoplasticum* with AgNP treatment (green). Control groups include naïve (black) and larvae injected with PBS 1× (gray).

## Discussion

The growing challenge of antifungal resistance has turned fungal infections into a major public health concern. These infections are marked by high rates of morbidity and mortality combined with the limited availability of effective drugs. This scenario highlights the need for new and effective antifungal treatments with alternative mechanisms of action to address these limitations. Moreover, understanding how these compounds work at a molecular level is essential for developing more effective therapies.^21,22^

The activity of AgNPs depends not only on the target organism but also on their synthesis method and intrinsic properties. AgNPs synthesized using waste-grass achieved 90% inhibition of *F. solani* at 20 μg/mL,^23^ while AgNPs biosynthesized using plant extract inhibited *Fusarium oxysporum* at concentrations of up to 100 μg/mL.^24^ In this study, AgNPs biosynthesized by *E. nigrum* showed complete inhibition of *F. keratoplasticum* growth at a concentration of 5.92 μg/mL. These results reinforce the potential of biogenic AgNPs biosynthesized by an extremophile fungus as an effective alternative for combating emerging fungal pathogens. Similar studies have demonstrated broad antifungal activity of AgNPs biosynthesized by *E. nigrum* against various fungi, including *Candida* spp., *Fusarium* spp.,^25,26^ and *Aspergillus* spp.^27^

To investigate the mode of action of AgNPs, *F. keratoplasticum* was exposed to biogenic AgNPs, and proteomic changes were analyzed by label free quantitative proteomics. The results revealed a significant increase in the abundance of proteins associated with oxidative stress responses including oxidoreductases, glutathione transferase, peroxidase, and superoxide dismutase. These enzymes play essential roles in detoxifying reactive oxygen species (ROS) and maintaining cellular homeostasis. AgNPs are known to induce ROS production, such as hydroxyl radicals, superoxide anions, and hydrogen peroxide, which can damage key cellular components, including lipids, proteins, and DNA.^18,28^ This suggests that oxidative stress is a central mechanism driving the antifungal activity of AgNPs.

Proteins involved in cellular respiration and transport, such as NADH-ubiquinone oxidoreductase and glutathione transferase, also showed an increased abundance. These proteins likely support energy-intensive processes, including xenobiotic efflux and extracellular electron transfer, which are critical for the cell’s response to stress. Similar proteomic patterns have been observed in *Candida parapsilosis* exposed to silver compounds, where proteins related to oxidative stress, detoxification, and cellular respiration were also increased in abundance.^29^

Enzymes such as glutathione transferase mitigate oxidative damage by conjugating glutathione to harmful intermediates, while superoxide dismutase converts superoxide radicals to less toxic hydrogen peroxide and molecular oxygen. Peroxidase enzymes further detoxify hydrogen peroxide by reducing it to water. However, the intense oxidative stress caused by AgNPs disrupts these pathways, leading to cellular damage and eventual death. These findings align with previous studies showing that AgNPs disrupt oxidative stress responses in *F. solani* ^30^ and impair plasma membranes and metabolic processes in *Fusarium graminearum*.^18^

Proteomic analysis also revealed a decreased abundance in proteins associated with essential metabolic pathways, particularly riboflavin biosynthesis and the electron transport chain (ETC). Riboflavin is a precursor of cofactors like FAD and FMN, which are essential for redox reactions.^26,31^ The observed reduction in proteins related to riboflavin biosynthesis suggests that the fungus ability to produce these cofactors is compromised, which may affect ETC efficiency and ATP production.^32,33^ One of the most significant advantages of AgNPs over conventional antifungal agents is their multimodal mechanism of action. Traditional antifungal drugs, such as azoles and echinocandins, target specific cellular processes (e.g., ergosterol synthesis or the fungal cell wall) and are often subject to the development of resistance.^34–36^ In contrast, AgNPs disrupt multiple cellular pathways simultaneously, including membrane integrity, protein folding, and energy metabolism, as demonstrated in this study. This mechanism of action reduces the likelihood of resistance development, making AgNPs a promising candidate for use in antifungal therapies, particularly in cases of drug resistant fungal infections.^37–39^

With the aim of determining the in vivo toxicity and activity of AgNPs, *G. mellonella* was used as a model to study whether AgNPs immune priming effect was induced.^40^ The use of *G. mellonella* larvae as a model organism is well established for evaluating the in vivo toxicity and antimicrobial activity of various compounds including AgNPs. This model has shown strong correlations with results obtained from vertebrate studies, making it a reliable and ethically favorable alternative.^20,41^ In the present study, AgNPs showed a favorable safety profile, as larvae exposed to the AgNPs exhibited no significant reduction in survival over 8 days. A rapid increase in hemocyte density was observed after AgNPs administration, suggesting activation of cellular immune responses. Proteomic analysis, however, revealed minor alterations, which reinforces the idea that biogenic AgNPs trigger a controlled or minor immune response without causing systemic toxicity. These findings align with previous studies showing that biogenic AgNPs synthesized by *F. oxysporum* were not toxic to *G. mellonella* larvae.^42^ Similarly, other biogenic AgNPs did not show toxicity to *G. mellonella*, endothelial cells, or human fibroblasts at concentrations between 0.1 and 1 μM.^43^

The antifungal activity of AgNPs was also assessed by infecting *G. mellonella* larvae with different doses of *F. keratoplasticum* conidia. Larval survival decreased significantly with increasing conidial doses, with 50% survival observed at 4 days for 10^4^ conidia, and total mortality occurring within 7, 5, and 3 days for doses of 10^5^, 10^6^, and 10^7^ conidia, respectively. Administering AgNPs 1 h after infection with 10^5^ conidia significantly delayed larval death, demonstrating the efficacy of AgNPs in mitigating fungal infection. These results are consistent with the protective effect reported for other metal-based compounds, such as gallium, which was shown to protect *G. mellonella* larvae from *Pseudomonas aeruginosa* infections.^44–46^

The antifungal potential of AgNPs demonstrated in this study is particularly relevant in the context of the growing problem of drug-resistant fungal pathogens. The development of alternative therapies with novel mechanisms of action is needed to address this challenge. AgNPs represent a promising solution due to their broad-spectrum activity and ability to target fungi through mechanisms, such as the induction of oxidative stress and structural disruptions. These nonspecific mechanisms are advantageous because they reduce the likelihood of resistance development. Moreover, the biogenic synthesis of AgNPs offers a sustainable and environmentally friendly approach, enhancing their safety and applicability. Their ability to activate immune responses without causing significant toxicity, combined with their effectiveness in controlling fungal infections in vivo, highlights their promise for clinical applications. Future studies should focus on elucidating their mechanisms of action in greater detail, optimizing their properties for improved efficacy and validating their use in more complex in vivo models. These efforts could establish AgNPs as a valuable tool in the fight against drug-resistant fungal infections.

## Conclusion

This study demonstrates the potential of biogenic AgNPs as effective antifungal agents against *F. keratoplasticum*, an emerging clinical pathogen. AgNPs inhibited fungal growth at low concentrations through mechanisms involving oxidative stress and metabolic disruption. Proteomic analysis highlighted changes in proteins related to the oxidative stress response and energy metabolism, confirming their antifungal activity. Using *G. mellonella* larvae as an in vivo model, this study provides preliminary evidence of low toxicity of biogenic AgNPs. Larvae exposed to AgNPs showed no signs of toxicity or activation of the immune system. Additionally, AgNPs delayed larval death in fungal infection experiments, showing their potential in reducing mortality caused by *F. keratoplasticum*. These findings align with studies showing the low toxicity and antimicrobial effects of the biogenic AgNPs. This work highlights the need for new antifungal therapies due to the rise of drug-resistant fungi. The multimodal action of AgNPs, involving oxidative stress and cellular disruption, makes resistance development more difficult compared with traditional antifungal drugs. Moreover, the biogenic synthesis of AgNPs enhances their clinical potential. Future studies should focus on improving AgNPs properties to increase their antifungal effects and validating their use in more complex in vivo models. Additionally, future studies are encouraged to include more detailed physicochemical characterization to better relate the nanoparticle properties to their biological effects. While promising, the safety and efficacy of biogenic AgNPs must be further validated in mammalian systems before they can be considered.

## Methods

### AgNP Biosynthesis and Characterization

AgNPs were biosynthesized using the cell-free culture filtrate of *E. nigrum* as previously described with some modifications.^16^ The endophytic fungus *E. nigrum* was isolated from the seaweed *Kallymenia antarctica* collected from King George Island 62° 22,793′ S 59° 41,813′ W and kindly donated by Dr. Hosana Debonsi (FCFRP-USP, Brazil). The preinoculum was cultivated on Sabouraud Dextrose Agar (SDA, Oxoid Ltd., UK) at 25 °C for 5 days. From SDA cultivation, four agar plugs (7 mm) were inoculated in 100 mL of rice broth (2 g L^–1^ parboiled rice powder (Camil Foods, Brazil) and incubated in a rotary shaker (Infors HT Ecotron, Switzerland) at 20 °C and 150 rpm during 6 days. Fungal biomass was harvested and washed three times with sterile distilled water. Biomass was incubated in distilled water at a 3:1 ratio at 25 °C, 150 rpm for 4 days. Fungal supernatant was collected by centrifugation (model 5702R, Eppendorf, Hamburg, Germany) and filtered through a 0.22 μm PVDF membrane. Silver nitrate (Sigma-Aldrich, Inc., St. Louis, MO, EUA) was added to *E. nigrum* filtrate in a final concentration of 1 mM. The mixture was heated at 70 °C for 5 min and stored in the dark at 25 °C. AgNPs biosynthesis was confirmed through color change of the mixture from light yellow to brown. The visualization of the surface plasmon resonance band was performed using a UV–vis spectrophotometer (PClass spectrophotometer, IMPLEN GmbH, Munich, Germany). The nanoparticles sizes, morphologies, and distribution was determined by transmission electron microscopy in a JEOL/JEM 2100 LaB6 200 kV instrument (JEOL, Boston, MA, USA).

### Fungal Strain and Conidia Production

*F. keratoplasticum* (ATCC 36031) was used in this study. A 100 μL of conidial suspension at 1 × 10^6^ conidia/mL was freshly subcultured on potato dextrose agar (Oxoid Ltd., UK) and incubated at 28 °C for 5 days. Conidia were collected using a sterile 0.9% saline solution and filtered through sterile Miracloth (MilliporeSigma, United States). The conidial suspension was adjusted to the required concentration for further analysis.

### Antifungal Activity of AgNPs Against *F. keratoplasticum*

The antifungal activity of AgNPs was determined using the microdilution broth method following the M38A2 protocol from the Clinical and Laboratory Standards Institute (CLSI, 2018). Briefly, in a 96-well polystyrene plate, 2-fold serial dilutions of AgNPs ranging from 0.01 to 5.82 μg/mL were prepared in RPMI 1640 (Sigma-Aldrich, EUA) buffered with 3-[*N*-Morpholino] Propanesulfonic Acid (MOPS) 0.165 M (Sigma-Aldrich, EUA). Conidia suspension was prepared in sterile 0.9% saline to a final concentration of 5 × 10^4^ conidia/mL. Saline 0.9% and RPMI 1640 were used as the negative control, and conidia suspensions with RPMI 1640 were used as the positive control. Plates were incubated at 37 °C for 48 h. The minimal inhibitory concentration was determined as the concentration that inhibits 90% (MIC_90_) of fungal growth. The experiments were conducted in triplicate with two biological replicates.

### *F. keratoplasticum* AgNP Treatment, Protein Extraction, and Purification

For the label-free quantitative proteomics experiments, *F. keratoplasticum* preinoculum was cultivated on PDA (Oxoid Ltd., UK) at 28 °C for 5 days. Subsequently, two agar plugs (7 mm) were inoculated into each of 10 Erlenmeyer flasks (150 mL) containing 50 mL of potato dextrose broth (PDB, Oxoid Ltd., UK) and incubated at 28 °C while being shaken at 150 rpm for 24 h to promote fungal biomass formation. Afterward, five flasks were kept as controls without AgNPs, while AgNPs at a concentration of 1.45 μg/mL were added to the remaining five flasks. The cultures were further incubated at 28 °C with shaking at 150 rpm for an additional 24 h. Biomass was collected using sterile Miracloth (Millipore Sigma, United States), and the wet weight was measured to ensure sample homogeneity.

To extract the protein, the *F. keratoplasticum* mycelia from each replicate were ground into a fine powder using a mortar and pestle with the aid of liquid nitrogen. For every gram of mycelium, 4 mL of lysis buffer (8 M urea, 2 M thiourea, and 0.1 M Tris–HCl, pH 8.0) containing protease inhibitors (aprotinin, leupeptin, pepstatin A (10 μg/mL)), and phenylmethylsulfonyl fluoride (1 mM) was added. The mixture was then sonicated using an ultrasonic homogenizer (Bandelin Sonopuls, Bandelin electronic, Berlin) at 50% power for 10 s, three times. The lysates were centrifuged at 14,500*g* for 10 min, and the supernatant was collected. Protein concentration was determined using the Bradford method, and supernatants containing 100 μg of protein were precipitated with ice-cold acetone at a ratio of 1:5 at −20 °C for 18 h.

Subsequently, the proteins were pelleted by centrifugation at 14,500*g* for 10 min. The acetone was discarded, and the pellets were air-dried to allow for complete evaporation of the acetone. Next, the pellets were resuspended in 25 μL of the resuspension buffer (the same composition as the lysis buffer but without protease inhibitors). The protein content was quantified using the Qubit quantification system (Invitrogen, Waltham, MA, USA) with 2 μL aliquots, while 20 μL of samples were mixed with 125 μL of 50 mM ammonium bicarbonate and 1 μL of 0.5 M dithiothreitol. The proteins were reduced at 56 °C for 20 min and alkylated with 2.7 μL of 0.55 M iodoacetamide in the dark at 25 °C. Following this, Trypsin (500 ng, Promega) and 1% (w/v) ProteaseMAX Surfactant Trypsin Enhancer (Promega) were added, and the proteins were digested for 18 h at 37 °C. Following this, 1 μL of trifluoroacetic acid (TFA) was added to stop the digestion, and the samples were incubated for 5 min at 25 °C. The samples were centrifuged at 14,500*g*, and the supernatants were purified using C-18 spin columns (Pierce, Thermo Scientific) following manufacturer’s instructions.

The samples were dried using a SpeedVac concentrator (Thermo Scientific Savant) for 4 h at 38 °C and resuspended in 2% (v/v) acetonitrile and 2% TFA. Two microliters of sample containing 750 ng were injected into a Q Exactive mass spectrometer (ThermoFisher Scientific) connected to a Dionex Ultimate 3000 RSLCnano chromatography system. Mass spectrometry and data analysis using MaxQuant software were performed according to previously described parameters.^47^

### AgNP Toxicity in *G. mellonella*

#### *G. mellonella* Larvae, Larval Culture, and Inoculation

*G. mellonella* larvae were purchased from Livefoods Direct Ltd. (Sheffield, UK) and kept at 15 °C to prevent pupation. To assess the toxicity of silver nanoparticles (AgNPs), groups of 10 healthy larvae in the sixth instar of development, weighing between 200 mg and 250 mg, were inoculated with 20 μL of each treatment using a U-100 insulin syringe (Terumo Europe, N.V., Belgium). AgNPs were administered in two different doses, 2.58 mg/kg and 1.29 mg/kg, which corresponds to AgNPs dispersions of 23.33 μg/mL and 11.66 μg/mL, respectively. PBS 1× was the negative control, and naïve larvae were used as the batch control. All groups were placed in 9 cm Petri dishes and incubated at 37 °C, and the survival rates of *G. mellonella* larvae were monitored over a 7 day period. Larval death was recorded when no movement or response to stimuli was observed. The experiments were performed on three independent days.

### Determination of Hemocyte Density

*G. mellonella* larvae were inoculated with 20 μL of AgNPs (2.58 mg/kg) and incubated at 37 °C for 0, 1, 3, 6, 16, 24, and 48 h. Empty microtubes and microtubes containing PBS 1× were kept on ice for hemolymph collection and immediate dilution, in order to prevent coagulation and melanization. After each time point, larvae (*n* = 3) were individually bled as previously described,^48^ to collect 40 μL of hemolymph, which was immediately diluted 1:5 in prechilled PBS 1×. As each larva was processed individually and dilution was performed promptly, no visible melanization or coagulation was observed. Hemocytes were counted by using a hemocytometer, and the density was calculated as the number of cells per mL.

### Proteins Extraction and Purification from *G. mellonella* Treated with AgNPs

*G. mellonella* larvae (*n* = 5) were injected with AgNPs (2.53 mg/kg), while PBS 1× served as the control. The larvae were incubated at 37 °C for 24 h. After incubation, the larvae were bled, and 120 μL of hemolymph was pooled, diluted 1:5 in PBS 1×, and centrifuged at 2500*g* for 10 min to obtain cell-free hemolymph. The entire process was carried out on ice to prevent hemolymph melanization. The hemolymph supernatant was collected and precipitated with 5 parts of acetone. Following precipitation, the protein content was quantified using the Qubit quantification system (Invitrogen, Waltham, MA, USA). Fifty-five micrograms of protein were digested and purified as previously described.^49,50^ The digested peptides (25 μg) were further purified using C18 spin columns (Pierce, Thermo Scientific) and dried in a SpeedVac concentrator (Thermo Scientific Savant) at 38 °C for 4 h. The samples were resuspended in 2% acetonitrile and 2% TFA, sonicated in a water bath for 5 min, and centrifuged at 15,500*g* for 5 min. The resulting supernatant was collected and used for mass spectrometry analysis.

A total of 600 ng of the peptide mixture was eluted and analyzed on a high-resolution QExactive mass spectrometer (ThermoFisher Scientific, USA), coupled to a Dionex Ultimate 3000 (RSLCnano) chromatography system. The peptides were separated via a gradient of increasing acetonitrile on a 50 cm EASY-Spray PepMap C18 column with a 75 mm diameter using a 133 min reverse-phase gradient at a flow rate of 300 nl min^–1^.

### Proteomic Data Analysis

Proteomic data analysis was performed using the Andromeda search engine integrated within the MaxQuant software (version 1.6.6.0).^51^ Predefined search parameters were adhered to ensuring consistency throughout the process.^41,47^ Further data processing, statistical evaluations, and graphical representations were achieved using Perseus version 1.5.5.3.^52^ Normalized LFQ intensity values were utilized to measure protein abundance, and contaminant proteins as well as peptides identified by sites were filtered out. The LFQ intensities were log_2_ transformed, and the samples were categorized into control and AgNP treatment groups. Proteins that were not detected in all three replicates from at least one group were excluded from the data set. Missing values were imputed with values simulating low-abundance proteins, selected randomly based on a distribution adjusted 1.8 times below the mean standard deviation, with a width of 0.3 times the standard deviation. Pairwise comparisons between two groups were performed using Student’s *t* tests with an FDR threshold of 0.05 on the postimputation data.

Volcano plots were created by mapping the log_2_ fold change values on the *x*-axis and the negative log *P*-values on the *y*-axis for each sample comparison. Proteins meeting the criteria of ANOVA (*P* < 0.05) and a fold change of ≥1.3 were considered SSDA and were used in subsequent analyses. The LFQ intensities were normalized by z-score for hierarchical clustering of the median values of SSDA proteins, with Euclidean distance used for clustering. To explore GO term enrichment in biological processes, cellular components, and molecular functions, a Fisher’s Exact test was applied with a Benjamini–Hochberg FDR cutoff of 5%.

The mass spectrometry proteomics data were submitted to the ProteomeXchange Consortium via the PRIDE partner repository under the identifier PXD060892.^53^

### Survival Analysis of *G. mellonella* Infected with *F. keratoplasticum*

To investigate the survival of *G. mellonella* larvae following *F. keratoplasticum* infection and to determine the optimal infectious dose (LD_50_), conidial suspensions were prepared at four concentrations: 10^4^, 10^5^, 10^6^, and 10^7^ conidia/mL. The conidial suspensions were diluted in sterile PBS 1×. Groups of 10 larvae, each weighing approximately 200–250 mg, were placed in 9 cm Petri dishes. In each group, larvae were injected with 20 μL of a conidial suspension using U-100 insulin syringes (Terumo Europe, N.V., Belgium) through the last right pro-leg. Two control groups were included: (i) naïve larvae (not injected) and (ii) larvae injected with PBS 1×. After inoculation, all larvae were incubated at 37 °C in the dark, and the survival rate was monitored daily over a period of 7 days. Larvae were considered dead when no movement or response to stimuli was observed.^46^

### AgNP Treatment of *G. mellonella* Infected with *F. keratoplasticum*

The AgNP treatment of *G. mellonella* larvae infected with *F. keratoplasticum* was evaluated. The selected dose of AgNPs (2.58 mg/kg) and the infection dose of *F. keratoplasticum* (10^5^ conidia/mL) were chosen based on two criteria: the ability of the nanoparticles to effectively combat *F. keratoplasticum* without causing toxicity in *G. mellonella*, and the survival outcomes observed in *G. mellonella* infected with *F. keratoplasticum*. Five experimental groups of *G. mellonella* larvae (*n* = 10) were established for this phase: (i) larvae infected with *F. keratoplasticum* (10^5^ conidia/mL) and treated with AgNPs (2.58 mg/kg) 1 h postinfection; (ii) larvae infected with *F. keratoplasticum* (10^5^ conidia/mL) and injected with PBS 1× after 1 h; (iii) larvae treated with AgNPs (2.58 mg/kg) and injected with PBS 1× after 1 h; (iv) control larvae injected twice with PBS 1×; and (v) naïve larvae (not injected). All injections were performed with 10 μL using U-100 insulin syringes (Terumo Europe, N.V., Belgium), and when two injections were administered, they were delivered in different pro-legs to minimize local trauma. All larvae were placed in 9 cm Petri dishes and incubated at 37 °C in the dark, and survival rates were monitored daily for 7 days. Mortality was determined based on the absence of movement or response to stimuli.^46^

### Statistical Analysis

All survival data were compiled and analyzed using Kaplan–Meier survival curves to visualize the differences among the various treatment groups. Pairwise comparisons between groups, such as infected larvae versus infected larvae treated with AgNPs, were performed by using the log-rank (Mantel–Cox) test to determine statistical significance. A *P*-value of less than 0.05 was considered statistically significant. Each experiment was independently repeated on three different occasions, and the results are expressed as the mean ± standard deviation. Statistical analyses were carried out using GraphPad Prism software (version 9.3.0).

## Supplementary Material



## Notes

*F. keratoplasticum* proteins ID, GO terms, and information from MaxQuant Analysis and *G. mellonella* proteins ID, GO terms, and information from MaxQuant Analysis (XLSX)

## Abbreviations

AgNPs: silver nanoparticles
PBS: phosphate buffered saline
LFQ-MS: label free quantification mass spectrometry
PCA: principal compound analysis
PDA: potato dextrose agar.
